# Solid–Liquid-Hysteresis Materials Based on Reversible Covalent Cross-Linking of Liquid Monomers

**DOI:** 10.1021/acspolymersau.5c00139

**Published:** 2025-12-09

**Authors:** Thomas Höfer, Albert Rössler, Oliver I. Strube

**Affiliations:** † Institute of Chemical Engineering, Universität Innsbruck, 6020 Innsbruck, Austria; ‡ ADLER-Werk Lackfabrik Johann Berghofer GmbH & Co KG, 6130 Schwaz, Austria

**Keywords:** polymers, cycloaddition, liquefication hysteresis, phase change material, covalent adaptable network

## Abstract

When a two-component liquid resin is cured, it usually ends up in the solid state and cannot be transferred to the liquid state again. This work introduces a polymeric material, which cross-links covalently at ambient temperatures, but can still be liquefied again on demand. The mechanism is based on the well-known concept of reversible cross-links, e.g., via Diels–Alder-reactions, which allow de-cross-linking at elevated temperatures. The presented material, however, differs from known variants by the fact that the non-cross-linked state is liquid at ambient conditions. Thus, the cross-linked solid material can be reversibly transferred to the liquid state again by heating. In contrast to classical melting, the material remains in the liquid state after cooling for a prolonged time. It therefore shows a behavior that is reminiscent of a melting hysteresis but is rather based on a superposition of thermodynamics and kinetics. At room temperature, cross-linking occurs rather slowly, while it is massively accelerated at raised temperatures. In contrast to supercooled liquids with a physical melting hysteresis, materials with a herein described “chemical liquefication hysteresis” are attractive for multiple applications, e.g., recycling, adhesives, coatings, or even polymeric bulk materials.

## Graphical abstract

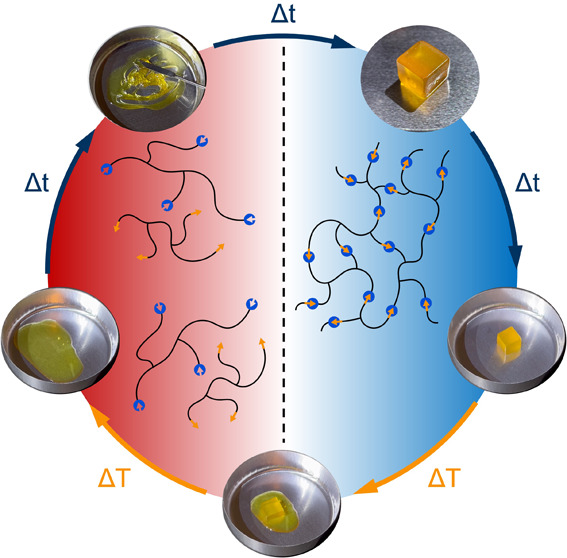

## 1 Introduction

Trivially, a solid material becomes a liquid if it is heated above its melting temperature. Likewise, if a liquid material is cooled below its freezing temperature, then it becomes solid. For most materials at standard conditions, melting and freezing temperatures are virtually equal. If these two values differ, melting hysteresis occurs, and the liquid is called supercooled.^1^ Besides a few exceptions,^2^ these liquids are metastable below their melting point and can easily be triggered for solidification by formation of crystal nuclei.^3,4^ Supercooled fluids might be used for a few special purposes, like supercooled ionic liquids in batteries^5^ or heat-free metal joining.^6,7^ In 2018, Barz and Sommer reported about modeling hysteresis in phase change materials for thermal energy storages, where hysteresis mainly occurs within mixtures of different polymers.^8^ Melting hysteresis might also occur due to surface effects, which was used by Martin et al. to design undercooled metal particles.^7^

In this paper, we describe a novel kind of thermally triggerable liquefication hysteresis, which allows feasible processing of the liquid state and potentially enables many technical applications for this kind of behavior. In contrast to the above-mentioned examples, the hysteresis is not enabled by physical properties like the melting point of a substance. Rather chemical reactions are responsible, in particular reversible cross-linking via covalent adaptable networks (CANs).

Countless review papers deal with self-healing or recyclability of cross-linked polymers based on CANs, which describe polymer networks, containing dynamic covalent bonds.^9–13^ One of the most frequently applied mechanisms in this field is the Diels–Alder (DA) cycloaddition between a diene and a dienophile and its reversible retro-Diels–Alder-reaction (rDA), respectively. This type of reaction was originally used as a protective group in organic synthesis.^14,15^ Meanwhile, it is frequently used in reversible polymer cross-linking. One major benefit of DA-reactions is the 100% atom economy. No coupling products are released during cross-linking. Accordingly, de-cross-linking can be triggered without the use of additional reagents. Furan-compounds as the diene and maleimide-compounds as the dienophile are used most frequently because of their thermodynamic equilibrium states. Furthermore, the equilibrium’s location can be influenced by varying the furan’s and the maleimide’s substituents, like calculated by Boutelle and Northrop,^16^ e.g., introducing the furan-functionality via 2-furfuryl alcohol or 2-furoic acid will yield different equilibrium states. With respect to this, the ideal DA-pair is chosen to have its equilibrium state at the cycloadduct’s side near room temperature (RT). At the same time, it is required to be almost completely reversible at temperatures below the start of irreversible side-reactions in the non-cross-linked state.

In 2021, Orozco et al. investigated the thermal stability and self-cross-linking of maleimide in furan/maleimide reversibly cross-linking polyketones. They demonstrated the irreversible self-polymerization of maleimide-groups under the formation of succinimide-monomers.^17^ This is in accordance with the results of Hopewell et al., who studied the homopolymerization of aromatic bismaleimides,^18^ and with McReynolds et al., who focused on the temperature-stability of an epoxy-based furan monomer in combination with bismaleimide-S (BMI-S).^19^ Cross-linking mainly occurred due to radical polymerization at 150 °C within three hours, which is in accordance with the results of Orozco et al.^17^ as well.

In 2023, van den Tempel et al. reported domino reactions in Diels–Alder polymer networks, named Double-Diels–Alder-reactions (DDA).^20^ It was shown that another furan-functionality can add to a furan/maleimide DA-adduct. In principle, this reaction is reversible, but temperatures of 180 °C or more are necessary, whereby side-reactions of maleimide-compounds become relevant.

Summarizing the relevant literature, the furan/maleimide DA-pair is highly suited to be used at moderate temperatures between RT and 150 °C. Furthermore, chemical equilibrium and reaction rate of DA-reactions are highly temperature-dependent, enabling sufficient control of the system. Radical polymerization of maleimide and DDA-reactions might be an issue for the longevity of the system.

There are two publications, distantly related to the material, aimed at this work. They are based on liquid monomers as well, but stimulation occurs via irradiation instead of temperature. Akiyama and Yoshida^21^ describe an UV-triggered, reversible process of liquid-crystalline, azo-group-functionalized, sugar-alcohol-based monomers. The material can be solidified as well as liquefied by irradiation with different wavelengths. In the second publication, Houck et al.^22^ describe a light-stabilized, reversibly cross-linkable material, which is in the liquid state at ambient conditions and solidifies under irradiation with green light. The material is solely solid when maintaining irradiation and liquefies within a few hours in the dark. The reversibility of the system is limited to approximately three cycles of solidification and liquefication. Irradiation-triggered CANs are always limited to the optical accessibility of the material.

However, to the best of our knowledge, all investigated thermally triggerable CAN-substances in the literature are solid in the non-cross-linked stage at RT. This makes thermal de-cross-linking and liquefication macroscopically appear like a physical melting process, similar to a thermoplastic material. If a system could be found that can be liquefied thermally but stays liquid under ambient conditions for a prolonged time, countless applications would be imaginable. Particularly noteworthy in this context are recycling, debonding on demand, or solvent-free one-component-coatings. All of these applications are already feasible via thermally activatable state-of-the-art materials, but they have to be processed under raised temperatures to avoid spontaneous and unintended solidification. This necessity could be circumvented by designing an RT-liquid monomeric system.

## 2 Experimental Section

### 2.1 Materials

β-Alanine (>98%; VWR), maleic anhydride (>98%; VWR), 2-furoyl chloride (>98%; Thermo Scientific), 2-furoic acid (>98%; Thermo Scientific), tetrahydrofuran (>99%; VWR; THF), acetic acid (>99%; VWR), ethyl acetate (>99%; Thermo Scientific), toluene (>98%; VWR), acetone (>99%; VWR), dichloromethane (>99%; VWR; DCM), sulfuric acid (96%; VWR), sodium hydrogen carbonate (>98%; VWR), a three-OH-functional ester-compound with an average molecular weight of 300 g mol^–1^, further just called “monomer” (“Capa 3031”, from caprolactone and trimethylolpropane; technical grade; Ingevity), and 4,4′-methylenebis­(*N*-phenylmaleimide) (95%; Thermo Scientific; BMI-S) were used as received. The ^1^H NMR-spectra (nuclear magnetic resonance) of all reactants can be found in the Supporting Information.

### 2.2 Synthesis of 3-Maleimidopropionic Acid

3-Maleimidopropionic acid was synthesized via a two-step synthesis. First, an intermediate was synthesized by reacting 40.00 g (1.0 equiv) of β-alanine with 48.43 g (1.1 equiv) of maleic anhydride in 400 mL of ethyl acetate/acetic acid (360 mL/40 mL) for 60 min under reflux. The precipitate was filtered off by suction, washed three times with 50 mL of ethyl acetate, and dried at room temperature (RT) to a constant weight. This reaction showed a quantitative yield. Subsequently, 73.90 g of the intermediate was refluxed in 350 mL of acetic acid for 120 min until the NH-signal in IR (infrared spectroscopy) at 3300 cm^–1^ disappeared. Acetic acid was removed with a rotary evaporator. The resulting mixture was extracted three times with 180 mL of toluene, and the extracted phases were dried in a rotary evaporator. 3-Maleimidopropionic acid was yielded as a white crystalline powder in 49% (^1^H NMR can be found in the Supporting Information). Further fractions were extracted with toluene, which were analyzed as partially polymerized 3-maleimidopropionic acid and therefore were discarded.

### 2.3 Preparation of Furan- and Maleimide-Functional Monomers

Furan-functional monomers were prepared from the OH-functional monomer (viscosity: 1600 mPas according to TDS) via the Einhorn-acylation. 10.00 g (1.0 equiv) of this monomer was reacted with 13.68 g (3.15 equiv) of 2-furoyl chloride in 60 mL of THF (tetrahydrofuran) with 8.46 mL of pyridine as a catalyst at RT. The reaction was completed by refluxing for 60 min. The precipitate was filtered off by suction and washed with THF, followed by removal of the filtrates’ solvent on a rotary evaporator. The oily crude product was dissolved in DCM and washed with 50 mL of a saturated sodium hydrogen carbonate solution and 50 mL of water. After removal of DCM in a rotary evaporator, a slightly yellow, viscous liquid was obtained in quantitative yield (viscosity: 17,556 mPas at 23 °C and 20 s^–1^; ^1^H NMR can be found in the Supporting Information).

Maleimide-functional monomers were prepared from 17.52 g (1.0 equiv) of the OH-functional monomer by esterification with 32.53 g of 3-maleimidopropionic acid (3.3 equiv) in 580 mL of toluene. As a catalyst, 1.32 mL of sulfuric acid was added. The mixture was refluxed for 30 min, using a Dean–Stark water separator. After being cooled to room temperature, the product was purified by washing the toluene phase with 250 mL of a saturated aqueous solution of sodium hydrogen carbonate twice. Toluene was removed from the organic phase on a rotary evaporator. The resulting viscous liquid was dissolved in acetone and filtrated, and the filtrate was dried in a rotary evaporator once more. The product was yielded as a colorless to slightly yellow, medium viscous liquid in 50% (viscosity: 2508 mPas at 23 °C and 20 s^–1^; ^1^H NMR can be found in the Supporting Information).

### 2.4 Viscosity Measurements

Viscosities of the monomers were measured on the instrument CAP 2000+ Viscosimeter (Brookfield), using a cone spindle CAP-09. The shear rate was set to 20 s^–1^ at a temperature of 23 °C.

### 2.5 ATR–FTIR-Analysis

IR measurements were performed in an attenuated total reflection (ATR)-setup (attenuated total reflectance) on the instrument “spectrum two FT-IR” (PerkinElmer). For monomer-characterization, all spectra were normalized to 75% transmittance of the carbonyl-signal around 1714 cm^–1^, using the equation below.

$$T_{normalized}\left(\mathrm{\nu ̅}\right)=100\cdot \left(1-0.75\right)\cdot \frac{100-T_{normalized}\left(\mathrm{\nu ̅}\right)}{100-T_{measured}\left({\mathrm{\nu ̅}}_{normalization}\right)}$$

*T*_normalized_—normalized transmittance (for a specific wavenumber). *T*_measured_—measured transmittance (for a specific wavenumber). ν—wavenumber/cm^–1^. ν_normalization_—wavenumber, chosen for normalization/cm^–1^.

Calculating the maleimide-conversion, the signal’s integral at 696 cm^–1^ was normalized to the integral of carbonyl at 1714 cm^–1^. The initial value, measured directly after the start of each experiment, was used as a reference for 0% maleimide-conversion. By this, information about the systems equilibrium states was obtained, and the cross-linking process could be tracked.

### 2.6 NMR-Spectroscopy

^1^H NMR and ^13^C NMR measurements were performed on a “400 MHz Bruker AVANCE 4 Neo” spectrometer. *d*_6_-DMSO, CDCl_3_, or D_2_O were used as solvents.

### 2.7 Thermal Cross-Linking, De-Cross-Linking, and Long-Term Equilibrium States

Furan- and maleimide-functional monomers were mixed in a stoichiometric manner and cured at different temperatures. Long-term experiments for cross-linking and de-cross-linking were performed on laboratory heating plates (Heidolph Instruments), adjusted to the target temperatures (40 °C, 60 °C, 80 °C, 100 °C, and 120 °C). Samples were placed onto the plate in an aluminum-cup. Surface temperatures were ensured by external measurement before an experiment. The maximum acceptable deviation from the target temperature was ±2 °C. For cross-linking in macroscopic experiments, a heating chamber (Binder ED-S 56; 62 L; natural convection) was preheated to the curing-temperature. De-cross-linking was performed either in the heating chamber or on a heating plate, depending on the experiment.

### 2.8 DSC-Analysis

To obtain information about the glass transition temperature of the polymer mixtures, differential scanning calorimetry-analysis (DSC) was performed (Netzsch; DSC 204 F1; aluminum crucibles). Two different mixtures were prepared: a stoichiometric mixture of furan- and maleimide-functional monomer (1:1) and a stoichiometric mixture in which 10% of the maleimide-functionalities were replaced by BMI-S (1:0.9:0.1). BMI-S was dissolved in the other monomers at 120 °C for approximately one minute, obtaining a clear, slightly yellow liquid. Both mixtures were cured at RT for 50 days, to be in a fully cross-linked state. Samples between 20 mg and 25 mg were measured from −20 to 120 °C at a heating rate of 5 °C min^–1^. Each sample was measured three times, yielding consistent results.

## 3 Results and Discussion

### 3.1 Synthesis and Characterization of the Functionalized Monomers

#### 3.1.1 Synthesis

A RT-liquid, OH-functional macromonomer was used as the basis for the furan- as well as the maleimide-functional component of the CAN (subsequently called “monomers”). Although their viscosities increase due to functionalization, the liquid characteristics of the monomers are still preserved. Figure 1 shows the complete reaction scheme for the synthesis of the furan- as well as the maleimide-functional monomer.

**Figure 1:**
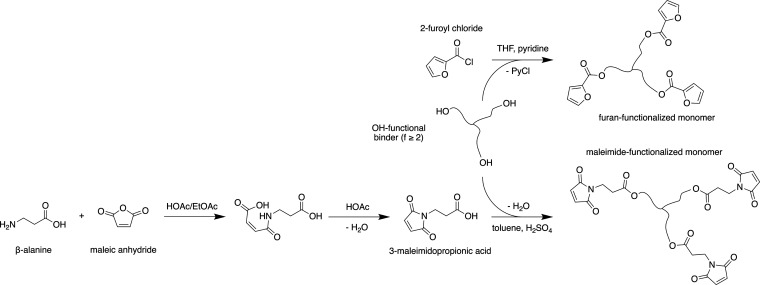
Overview of the synthesis of furan- and maleimide-functional monomers from a three-OH-functional monomer.

The furan-functional monomer was synthesized in one-step by esterification of the three-OH-functional monomer with commercially available 2-furoyl chloride. Alternatively, esterification was performed in toluene/H_2_SO_4_, using 2-furoic acid (61% yield), but the product showed a severe dark color.

To receive the maleimide-functional monomer, a three-step synthesis was chosen. β-Alanine and maleic anhydride were reacted to form an adduct, which was further converted into the intermediate 3-maleimidopropionic acid. In the last step, 3-maleimidopropionic acid was reacted with the three-OH-functional monomer.

In general, every OH-functional molecule (monomeric or polymeric) can be functionalized by the presented process. Only monomers with three or more functionalities will lead to elastomeric or thermoset networks, which are aimed to be obtained in this work.

#### 3.1.2 IR- and NMR-Spectroscopic Analysis

Both monomers were characterized by IR-spectroscopy. Figure 2 shows that the monomer has a strong absorption at around 3370 cm^–1^ (ν_OH_), owing to the three OH-functionalities. Neither the furan- nor the maleimide-functional monomer shows any sign of the original OH-signal, indicating full conversion. The furan-moiety of the furoate-monomer gives a characteristic signal around 1575 cm^–1^, which is attributed to aromatic ring absorption.^23^ The maleimide-monomer shows a characteristic absorption around 696 cm^–1^, due to ring bending of the unsaturated, five-membered ring.^24^

**Figure 2:**
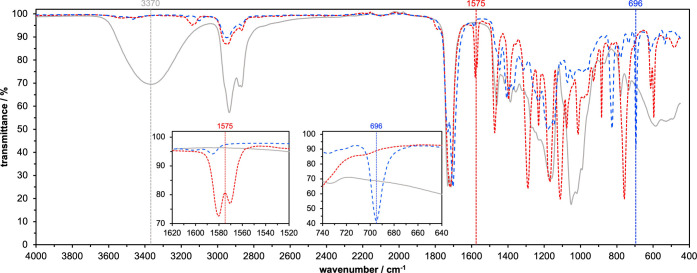
IR-spectra of the three-OH-functional monomer (solid gray), the furan-functional monomer (dashed red), and the maleimide-functional monomer (dashed blue). The most characteristic absorption band for each spectrum is highlighted.

Functionalization was further proven by NMR-spectroscopy. However, the used technical grade resin consists of different structural isomers. Hence, the spectra of the OH-functional monomer, as well as the furan- and maleimide-functional monomers, show a variety of multiplets and can thus not be interpreted quantitatively. Nevertheless, the appearance of furan and maleimide moieties in the respective monomers can definitely be observed, by comparing the spectra of reactants and products. The spectra and interpretation can be found in Figure S1 in the Supporting Information.

#### 3.1.3 Temperature Stability

To exclude falsification during cross-linking-measurements by decomposition of the monomers, thermal stability of both monomers was investigated via IR-spectroscopy and optical analysis. As visible in Figure S2a, the maleimide-functional monomer shows no significant changes in the IR-spectrum within 30 min at 120 °C. With progressive time, however, maleimide starts to self-polymerize, becomes solid, and its color changes from colorless to yellow. This process is irreversible and thereby undesired. At the same conditions, the furan-functional monomer was stable for several hours (Figure S2b), but it showed discoloration from yellow to brownish, which is attributed to residual impurities from the synthesis. Pictures of these measurements can be found in Figure S3. As a consequence, prolonged heating at high temperatures must be excluded. This is, however, not at all necessary, as the de-cross-linking kinetics is very fast at this point.

### 3.2 Mechanism of Chemical Hysteresis

By using monomers with CAN functionality that are liquid at RT initially, we aim to yield a solid thermoset comparable to a two-component adhesive. Beyond that, the system is supposed to be liquefiable again at elevated temperatures and to stay in this liquid state after cooling to RT again for a substantial time frame. Figure 3 illustrates this process on a molecular level. At low temperatures, the reaction is expected to be rather slow, while the equilibrium is on the polymeric side. With increasing temperature, reaction kinetics becomes faster, but the equilibrium is shifted more and more toward the monomers. Deduced, from a macroscopical point of view, we expect a fast liquefication at higher temperatures and a significantly slower solidification at RT. Thereby, a thermally triggered solid–liquid hysteresis can be achieved, and a wide field of technical materials processing becomes accessible.

**Figure 3:**
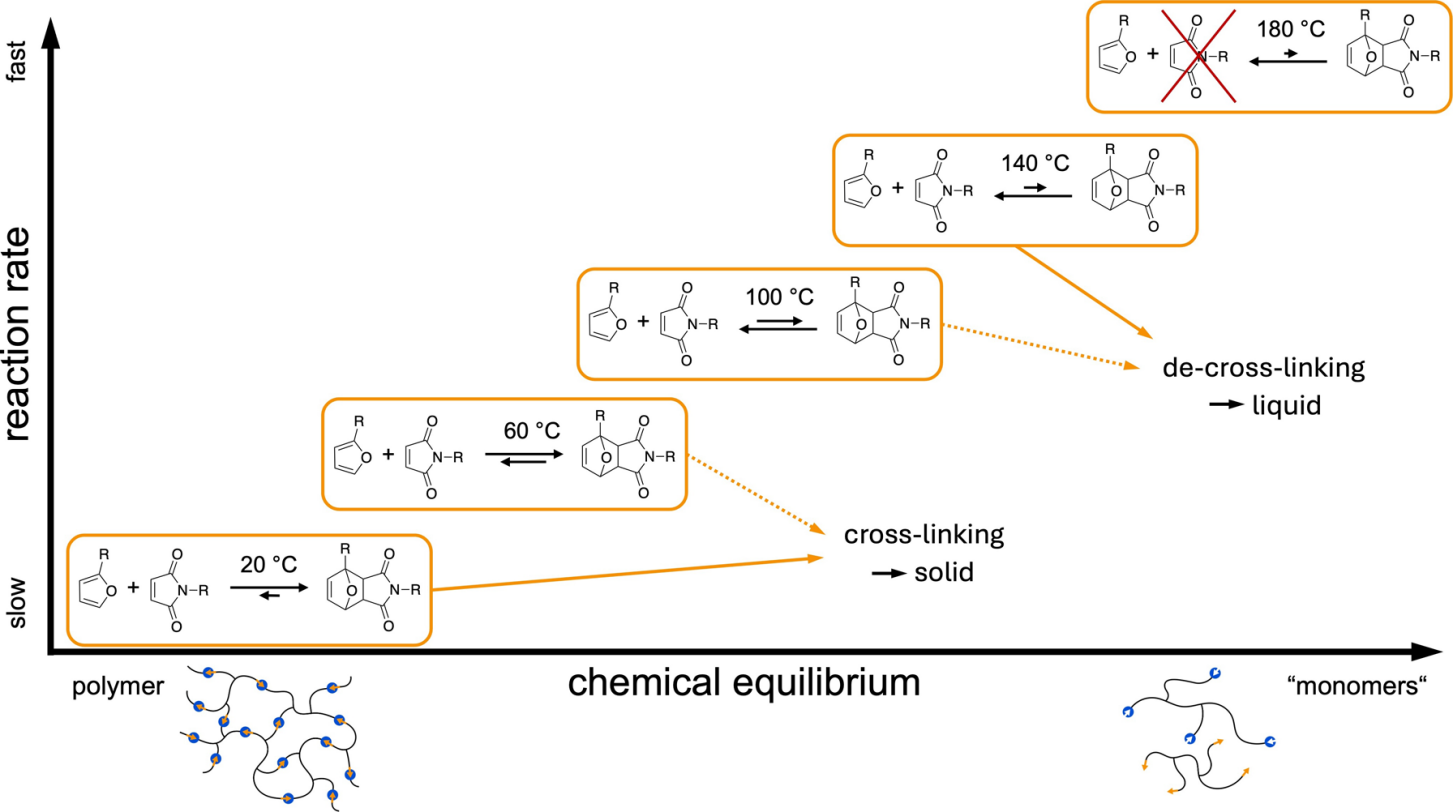
Expected dependence of chemical equilibrium and reaction rate as a function of temperature. Lower temperatures are aimed to be used for cross-linking, higher temperatures for de-cross-linking. At temperatures beyond 140 °C, the maleimide compound becomes instable.

### 3.3 Equilibrium States of the DA-System

A very important aspect in DA-systems is knowledge about the equilibrium at different temperatures. On the one hand, the equilibrium’s location is important to yield either a polymeric (solid; thermoset) or a monomeric (liquid) system. On the other hand, knowledge about the time to reach this equilibrium is likewise important. For this purpose, long-term experiments, followed via IR-spectroscopy, were performed with a stoichiometric mixture of the functional monomers. Figure 4 shows a reduction of the maleimide-signal as a function of the experiment’s first 20 days at different temperatures. The values of the normalized maleimide-integral can, however, not be strictly defined as chemical conversion of maleimide, as the DA-cycloadduct shows absorption close to the maleimide-band at 696 cm^–1^. This might possibly lead to lower maleimide-integrals, compared to the actual conversion. Thereby, in the following figures, the integral is depicted, instead of the conversion. Nevertheless, semiquantitative results can be yielded and a meaningful comparison between the different temperatures can be made. Unfortunately, other methods like NMR will yield even more distorted results, due to the usage of solvents, and thereby highly varying kinetics and thermodynamics of the system during the curing process.

**Figure 4:**
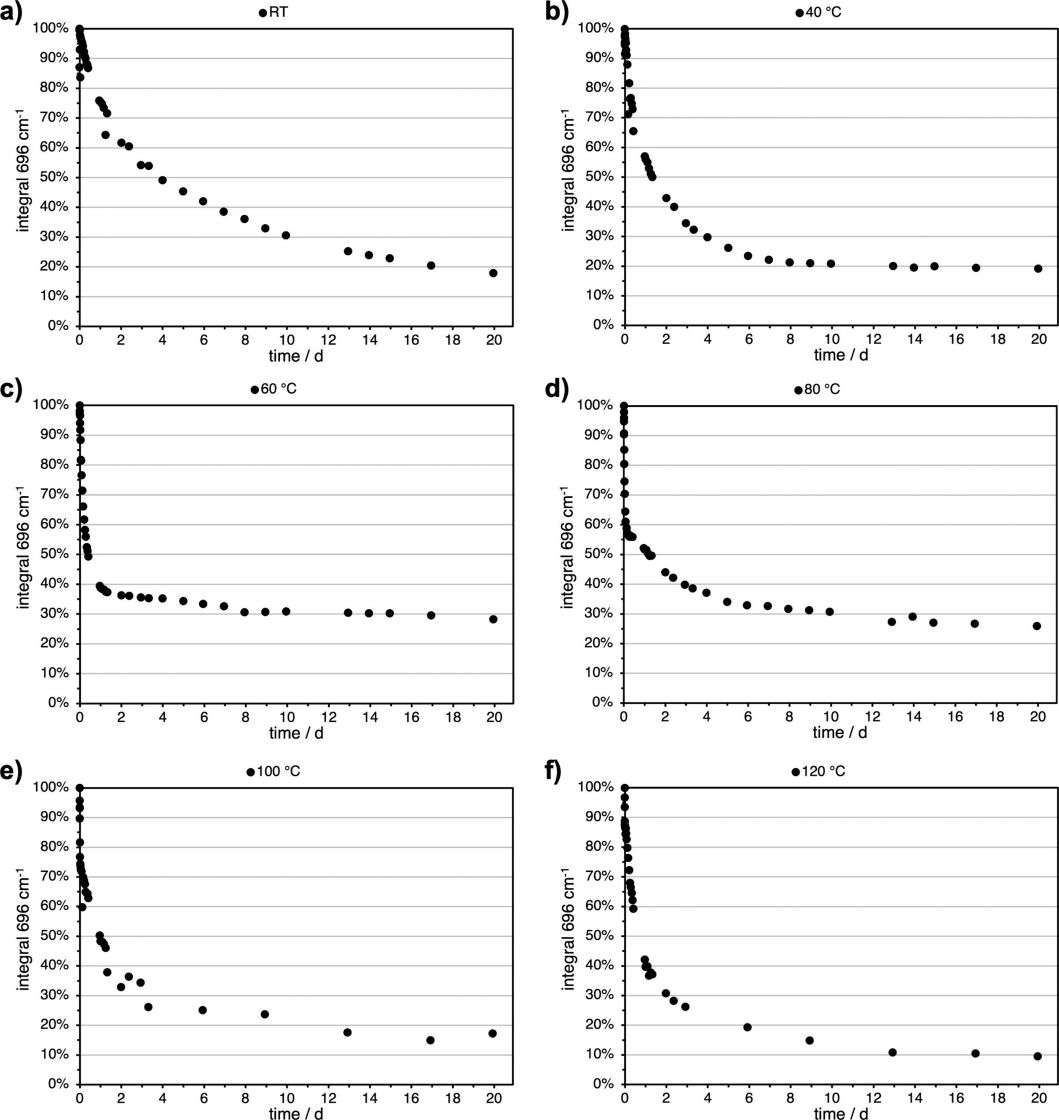
Reduction of the maleimide-signal at 696 cm^–1^ in the stoichiometric mixture of furan- and maleimide-functional monomers at RT (a), 40 °C (b), 60 °C (c), 80 °C (d), 100 °C (e), and 120 °C (f).

In Figure 4, it is recognizable that increased temperatures shift the equilibrium toward the maleimide-component and consequently toward the monomers. This was expected, as schematically indicated in Figure 3. Unexpectedly, at 60 °C, the maleimide-signal starts to be further reduced after reaching its equilibrium. At 80 and 120 °C, this effect becomes ever stronger. At 120 °C, it appears that the trend regarding the equilibrium is no more valid.

However, a focus on different time scales of the same experiment (Figure 5) shows that there are indeed equilibrium states at each temperature (black bullets). Locations of the equilibria and the time to reach them are listed in Figure 5g. As expected, the equilibrium shifts toward higher maleimide-signals with increasing temperatures. In contrast to Figure 4, it is now visible that equilibrium is already reached within very short times at higher temperatures.

**Figure 5:**
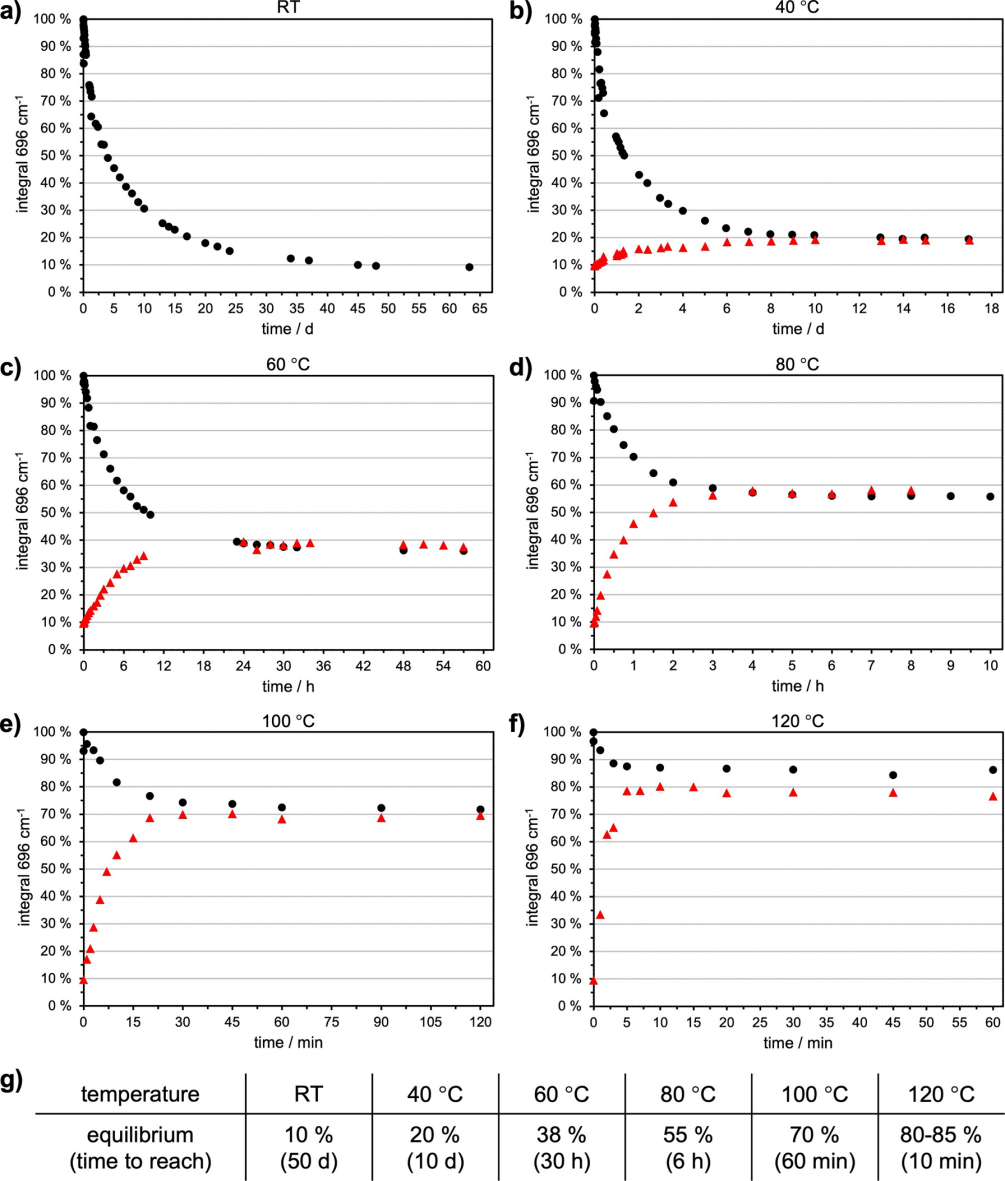
Zoom to equilibrium states at different time scales as a function of the temperature (a–f). Starting from the non-cross-linked monomers (black bullets) and from the cross-linked polymer (red triangles). Locations of equilibrium states as a function of temperature as well as the required times to reach them are shown in (g).

Subsequently, the maleimide-signal is further reduced by side reactions that follow different kinetics and are much slower than the DA-reaction itself. According to the literature, two major reactions are assumed to occur. The first and most important one is the radical homopolymerization of the maleimide-component.^17^ Hence, maleimide is irreversibly removed from the equilibrium, which favors the rDA-reaction. This results in further maleimide groups, which can polymerize. Another side reaction, occurring in lower extent, might be a DDA-reaction, where a second furan group adds to the furan-/maleimide-cycloadduct.^20^ These side-reactions were also apparent on the macroscopic scale, where viscosity raised continuously, until the resulting polymer became hard and brittle (comparable to Figure S3). As irreversible processes, they must be avoided to keep the system reusable for multiple cycles.

After 48 days at RT, the sample from the cross-linking experiment (Figure 5a) was heated to trigger the rDA-reaction for liquefication. Temperatures were chosen identical to the cross-linking-experiment, discussed above. The red triangles in Figure 5 show the results of these measurements. The rDA-reaction starts from a total maleimide-signal of 9%, which raises toward the same equilibrium states, compared to the cross-linking-experiment. The positions of the equilibrium states and the times to reach them are listed in Figure 5g. Named explicitly, they are located at 20% (40 °C; 6 d; b), 38% (60 °C; 30 h; c), 55% (80 °C; 6 h; d), 70% (100 °C; 1 h; e), and 80–85% (120 °C; 10 min; f). The equilibrium states are almost identical, whether coming from the non-cross-linked monomers or from the cross-linked polymer. Solely, the data at 100 (very slightly) and 120 °C (more significantly) differ. This might be attributed to side reactions as described above. Nevertheless, the signals are stable over the depicted time, indicating only a very low extent of these side reactions.

The results prove an almost complete reversibility and show that irreversible-side reactions are negligible during the cross-linking process at RT, as well as during the de-cross-linking process until the equilibrium is reached. Side-reactions occur only if higher temperatures are applied for prolonged times, which need to be much higher than necessary for reaching the equilibrium. Complementary IR-spectra taken directly after mixing the two monomers, after cross-linking for 48 d at RT, and after de-cross-linking for 10 min at 120 °C are shown in Figure S4.

### 3.4 Macroscopic Appearance of the Liquefication Hysteresis

To make the equilibrium states visible on the macroscopic scale, a cube of 1 cm^3^ was formed from a mechanically improved mixture of furan- and maleimide-functional monomers with BMI-S (1:0.9:0.1). The DSC-measurement revealing the cured polymers’ *T*_g_ of 21 °C can be found in Figure S5. Using this mixture, an illustrative experiment was performed to demonstrate the cyclical usability of the material. Figure 6 presents an exemplary selection of process parameters suitable for reversible cross-linking and de-cross-linking. Initially, the viscous liquid was poured into a cubic mold. After curing for 22 h at 50 °C and 6 days at RT, a solid cube was obtained (Figure 6a). Next, the rDA-reaction was triggered by heating the sample to 130 °C (Figure 6b–d). The cube completely liquefied within 5:30 min. Nevertheless, taking the insights from Section 3.3. into account, it was further heated for 11 min in total, to achieve complete de-cross-linking. After cooling to RT for 20 min, the viscous, liquid monomer mixture was regained (Figure 6e). In a cyclic manner, the sample was cured again in the cubic mold, followed by another round of liquefication. The macroscopic properties of the reliquefied system were highly comparable to the initial liquid system. Two video files, showing the liquefication step, respectively the macroscopic hysteresis appearance of the liquefied state, can be found in the Supporting Information.

**Figure 6:**
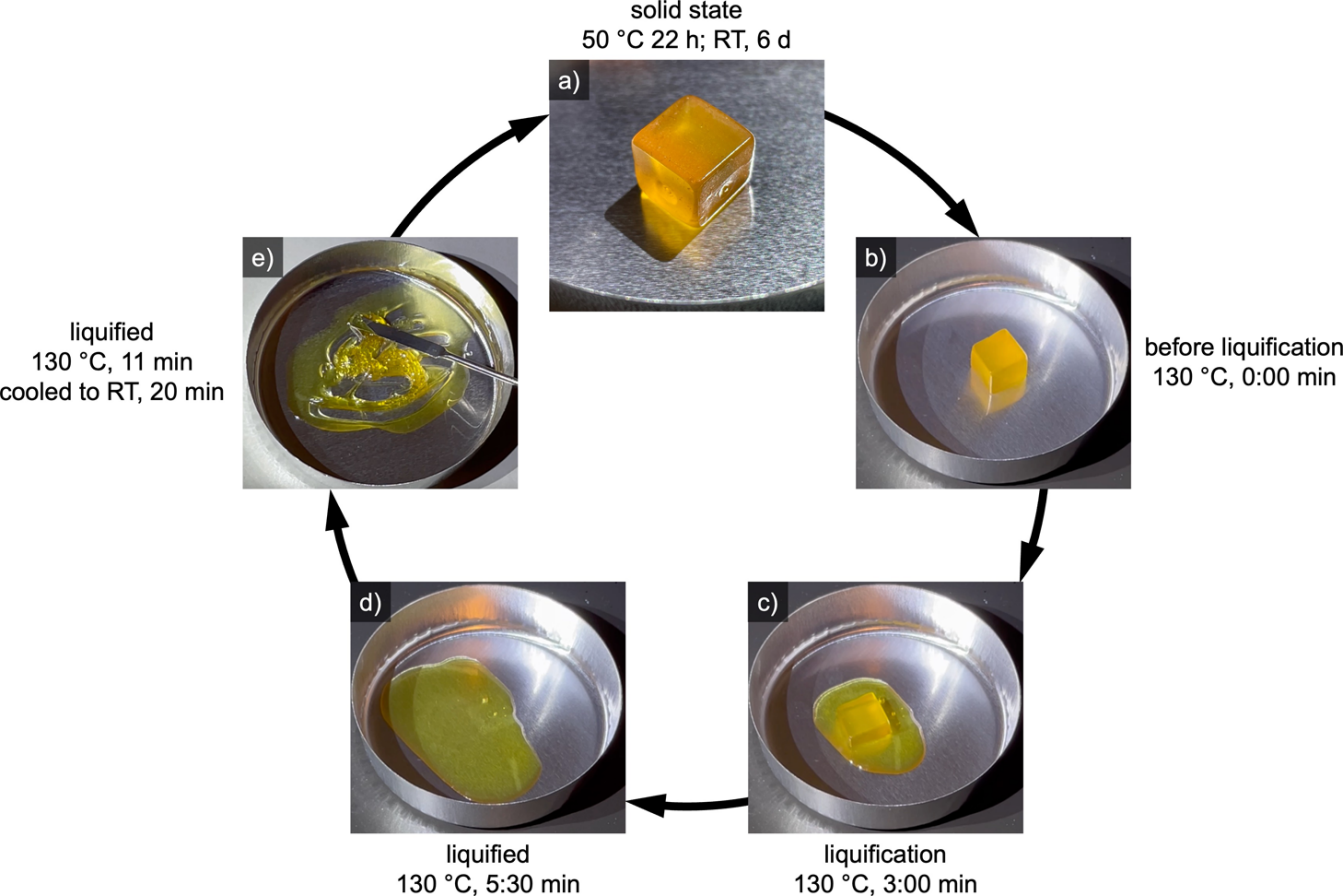
Macroscopic observation of the cyclic process, using a 1 cm^3^ cube at 130 °C. Starting from the solid state (a), the cube was liquefied at 130 °C (b–d), followed by cooling to RT (e). The system was cured again, to restart the cycle (a).

The experiment shown in Figure 6 was tracked via IR-spectroscopy for three cycles. In each of the cycles, slightly different conditions were chosen to investigate the stability of the system toward varying process parameters. In the first cycle, curing was performed at 50 °C for 22 h, followed by liquefication at 100 °C. In the second cycle, 50 °C for 22 h and 6 d at RT were used for curing, while liquefication was triggered at 130 °C for 10 min. The third cycle was similar to the second, but curing was extended to 24 h at 50 °C and 12 d at RT. De-cross-linking was performed once more at 130 °C. Cross-linking-conditions, de-cross-linking-conditions, and the maleimide-signals in the cross-linked and the de-cross-linked state can be found in Figure 7a. The progression of the maleimide-signal in IR is shown in Figure 7b. Below the dashed line at 35%, the system can qualitatively be referred to as “solid” with different degrees of hardness. Above this threshold, liquid properties with different viscosities are predominant.

**Figure 7:**
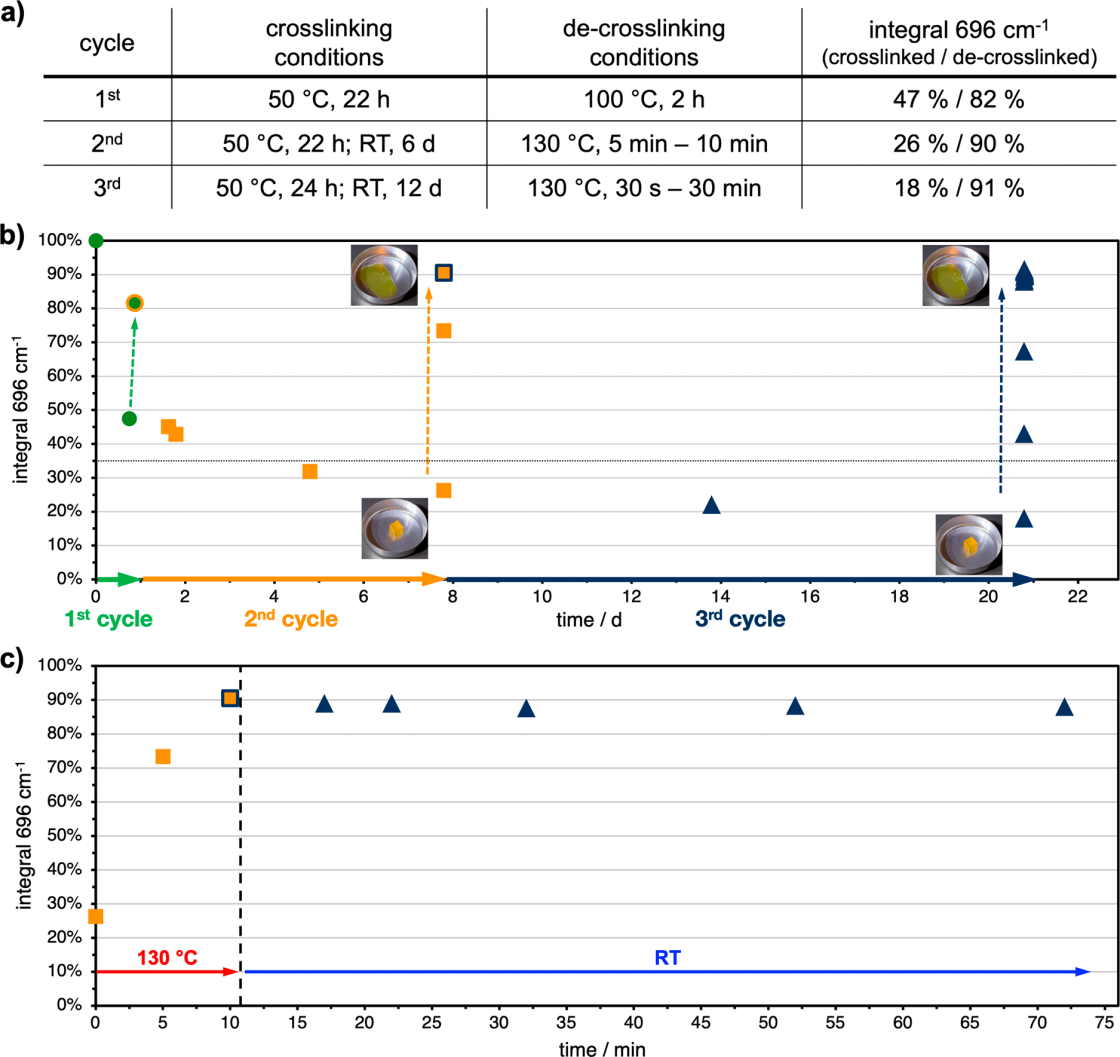
Tracking of the cyclic curing and liquefication process via IR-spectroscopy. Three cycles were performed with the same sample, using different conditions (a). The samples were cured and liquefied three times (b). Chemical hysteresis keeps the material in a liquid, de-cross-linked state for several hours (magnification of the transition from the second to the third cycle during cool-down at RT) (c).

After the third cycle, 91% of the maleimide-signals were regained within five minutes, which is identical to the second cycle. Furthermore, the maleimide-signal remained constant at 90 ± 2% for 30 min at 130 °C (Figure S6). Hence, it can be concluded that side reactions only occur to a negligible extent within the liquefication step. This confirms the cyclic reusability of the polymer system, as well as the system’s stability toward the processing temperatures for liquefication, which turned out to be ideally 130 °C for 10 min. The liquefication hysteresis is further highlighted in Figure 7c, where the maleimide-signal is tracked during cool-down at room temperature for 60 min. Within this window, a constant maleimide-signal between 90% and 88% was observed. This very slow decrease kept the system in a liquid state for a substantial amount of time. Macroscopically, the material was observed to remain in a processable state for several hours to days. These results are in accordance with the findings from Section 3.3, as the cyclical experiments are a combination of the previously described equilibrium states. Slightly different kinetics and thermodynamics can be explained by the addition of BMI-S, which influences the reactivity and *T*_g_ of the mixture.

Summarizing these results, a chemical hysteresis occurs due to a very slow cross-linking process at RT, combined with a very fast de-cross-linking process at 130 °C. Macroscopically, this effect appears like a massive “melting” hysteresis, opening a wide field of technical applications.

Though the described material is based on a specific monomer, this basis should, in principle, be highly tunable. The mixture described in this paper could be mechanically adapted by addition of different amounts of BMI-S, but also other comonomers (liquid or solid) could be added. Miscibility or solubility of the monomers and liquidity of the mixture at RT are the only requirements in the selection of comonomers. A certain amount of viscosity is actually beneficial for, e.g., adhesive systems to facilitate application. Also, hot pressing is possible to ensure good adhesion. If the system becomes too viscous, e.g., in coating applications, small amounts of solvents, such as butyl acetate, might be added without causing significant issues.

## 4 Conclusions

Based on low-cost reactants, furan- and maleimide-functional liquid monomers were synthesized by a straightforward esterification process. These two monomers were mixed in a stoichiometric manner and investigated in long-term experiments at different temperatures for cross-linking. The change of the maleimide-signal was tracked via IR-spectroscopy. Between RT and 120 °C, huge differences in the reaction rate and chemical equilibrium states of the cycloaddition were observed. At RT, setting of the equilibrium at 9% maleimide-signal required 50 days, while at 120 °C, the equilibrium was located around 85% and reached within 10 min. These different equilibrium states were used for solidification and liquefaction of the monomer mixture. The fast liquefication appeared highly similar to a physical melting process, while solidification of the RT-liquid monomers was negligible at RT for several hours. Due to these effects, a material with a kind of “chemical melting hysteresis” was obtained. This hysteresis between “melting” and “freezing” is highly temperature- and time-dependent and is connected to the kinetics and equilibrium states. Furthermore, the mechanical properties of the cured material can be controlled by addition of comonomers. E.g., by dissolving small amounts of the rigid monomer BMI-S within the liquid monomers, *T*_g_ and macroscopically observed hardness could be raised significantly. Finally, the system was shown to be reversible multiple times, enabling cyclic reuse of the material, e.g., in coating applications or adhesive technology. This paper focuses on DA- and rDA-reactions, but in principle, the concept should work with every kind of reversible reaction and is not limited to a specific type. The crucial aspect is to have RT-liquid monomers and sufficiently temperature-dependent kinetics and thermodynamics.

## Supplementary Material







## Data Availability

The data of this study are available from the authors on request.

## Notes

^1^H NMR spectra of all raw materials and purified products, thermal stability of the monomeric compounds and the mixture, IR-spectra of the reversible process, and DSC-measurements (PDF)
Liquefication of the cube, according to Figure 6 in Section 3.4; 20-times faster (MP4)
Hysteresis behavior of the cube after 20 min at RT, according to Figure 6 in Section 3.4 (MP4)
