# Supplementary material for: Solid–Liquid-Hysteresis Materials Based on Reversible Covalent Cross-Linking of Liquid Monomers
Source: ACS Polym Au. 2025 Dec 9;6(1):315–24. doi: 10.1021/acspolymersau.5c00139 (PMC12903472; doi:10.1021/acspolymersau.5c00139)
Supplement: Supplementary file 1 [file lg5c00139_si_001.pdf]

Solid-liquid-hysteresis materials based on reversible covalent crosslinking of liquid monomers

Supporting Information

Thomas Höfer, Albert Rössler, and Oliver I. Strube*

Affiliation:

T. Höfer, O. I. Strube:
Institute of Chemical Engineering
Universität Innsbruck
6020 Innsbruck, Austria
E-Mail: oliver.strube@uibk.ac.at

A. Rössler:
ADLER-Werk Lackfabrik Johann Berghofer GmbH & Co KG
6130 Schwaz, Austria
E-Mail: albert.roessler@adler-lacke.com

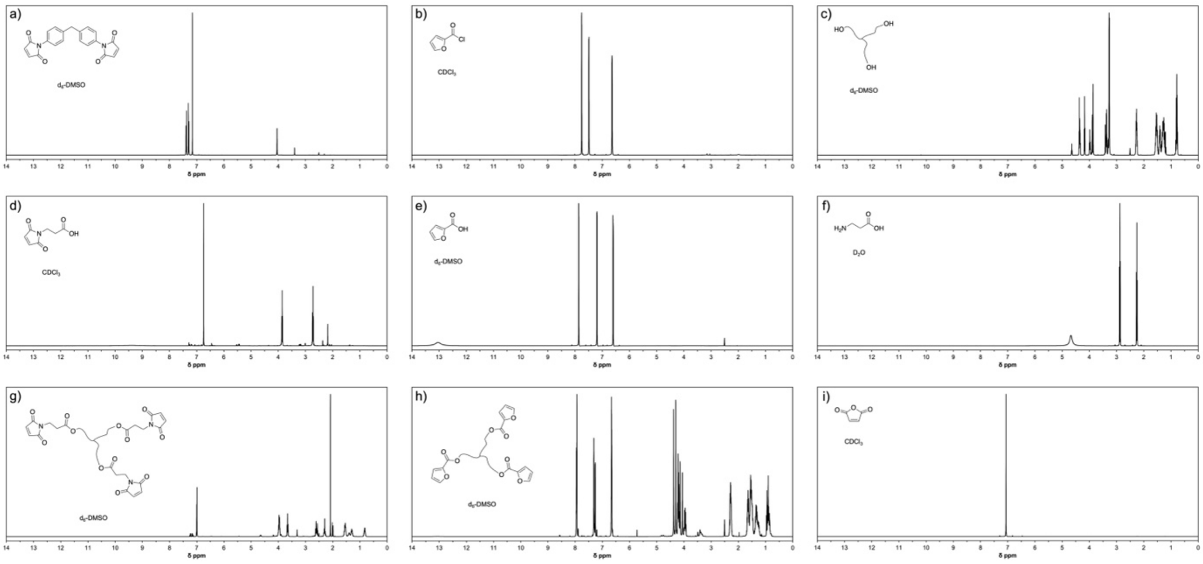

Figure S1. ^{1}H-NMR-spectra of raw materials and purified products in appropriate solvents. Bismaleimide S (a), 2-furoyl chloride (b), OH-functional monomer (c), 3-maleimidopropionic acid (d), 2-furoic acid (e), β-alanine (f), maleimide-functionalized monomer (g), furan-functionalized monomer (h), maleic anhydride (i). Characteristic furan-signals: 6.7 ppm, 7.3 ppm, 7.9 ppm (all dd). Characteristic maleimide-signal: 7.0 ppm (s)

Figure S1 shows the ^{1}H-NMR-spectra of the non-functionalized monomer (c), the furan-functionalized monomer (h), and the maleimide-functionalized monomer (g). Whereas the non-functionalized monomer shows no signals at 5 ppm or higher, the spectrum of the furan-functionalized monomer reveals three peaks at 6.7 ppm, 7.3 ppm, and 7.9 ppm, which are attributed to the three hydrogen atoms of the furan-ring. The maleimide-functionalized monomer shows a peak at 7.0 ppm, due to the two maleimide-protons. In contrast to the singlet in 3-maleimidopropionic acid, the signal is split into two peaks, which is attributed to different chemical environments of the protons after esterification (coupling constant J = 3.7 Hz). These characteristic peaks are also present in the spectra of 2-furoic acid (e; three times dd) and 3-maleimidopropionic acid (d; singlet), respectively. The chemical shifts of the maleimide-peaks are slightly different, owing to the use of various solvents ($CDCl_3$ for 3-maleimidopropionic acid and d_6-DMSO for the maleimide-functionalized monomer).

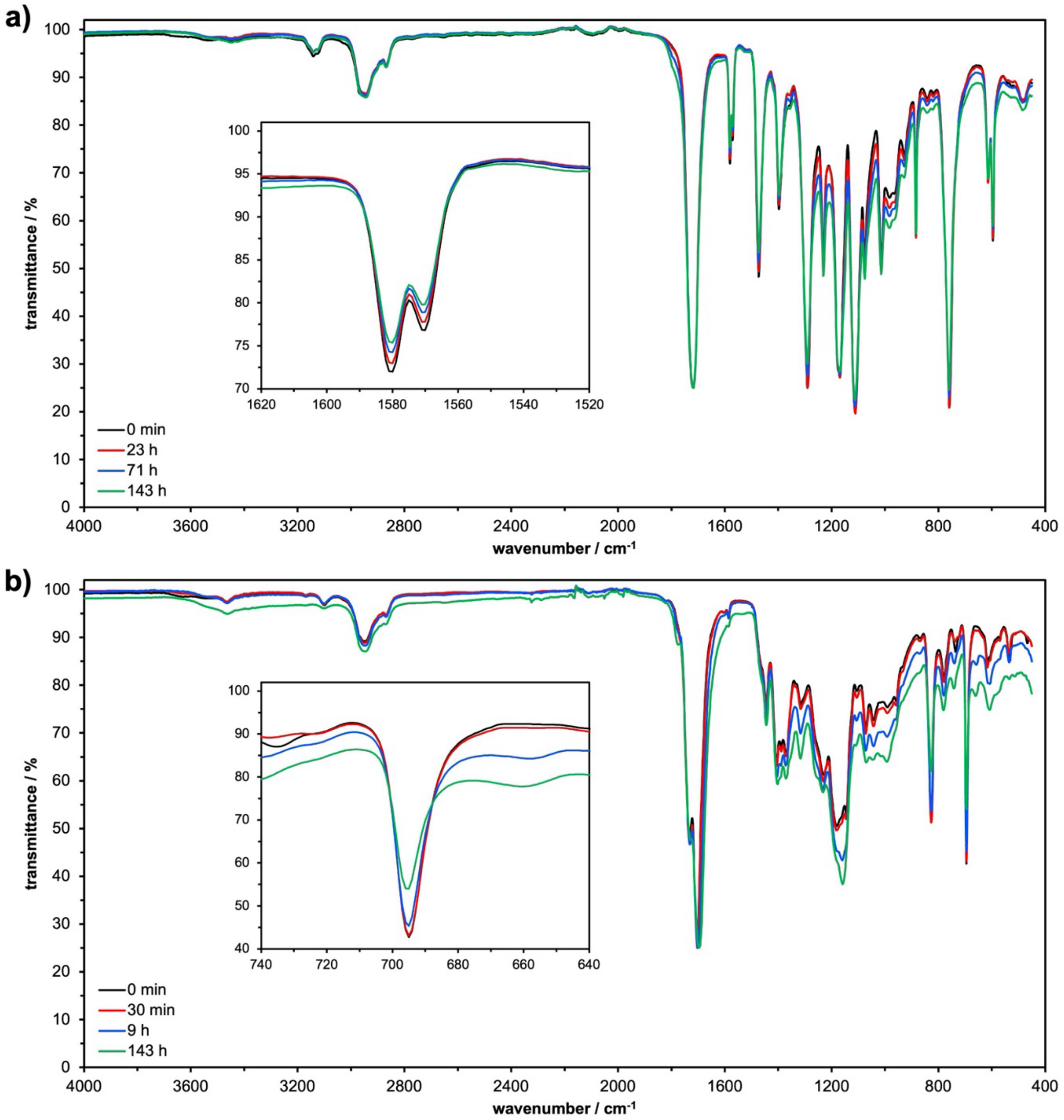

Figure S2. IR-spectra of the furan- (a) and the maleimide- (b) functionalized monomer at 120 °C and different times. The maleimide's transmittance at 696 cm^{-1} is highlighted.

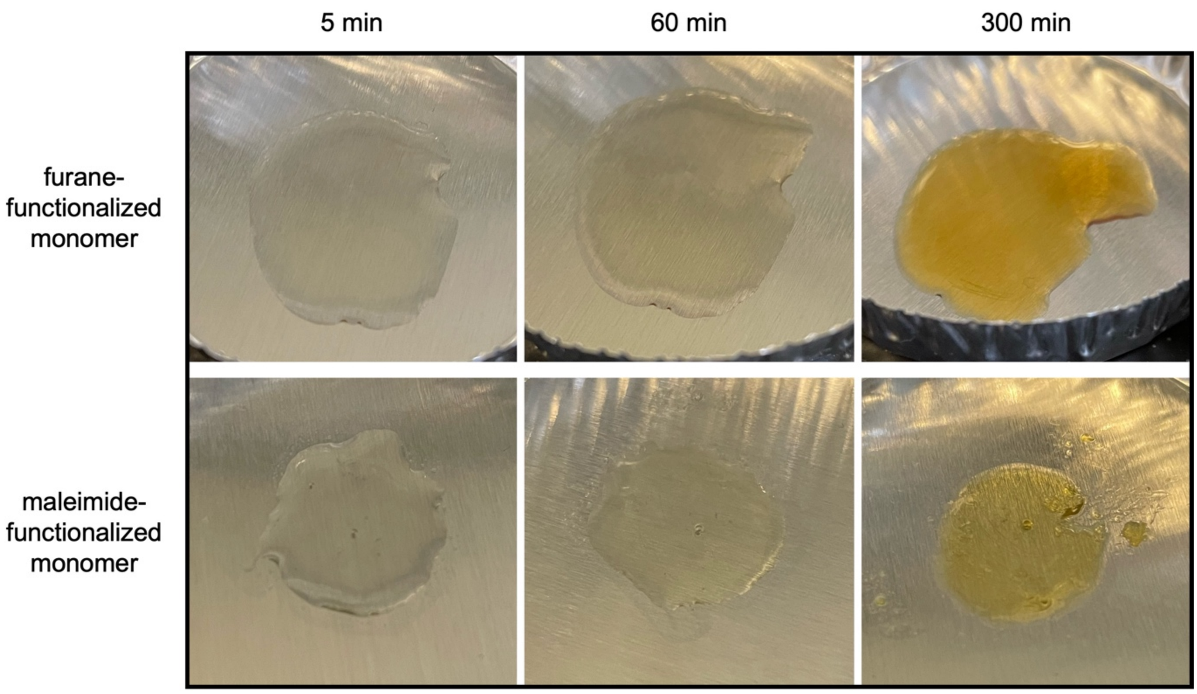

Figure S3. Optical tracking of the furan- and the maleimide-functionalized monomer's temperature stability at 120 °C.

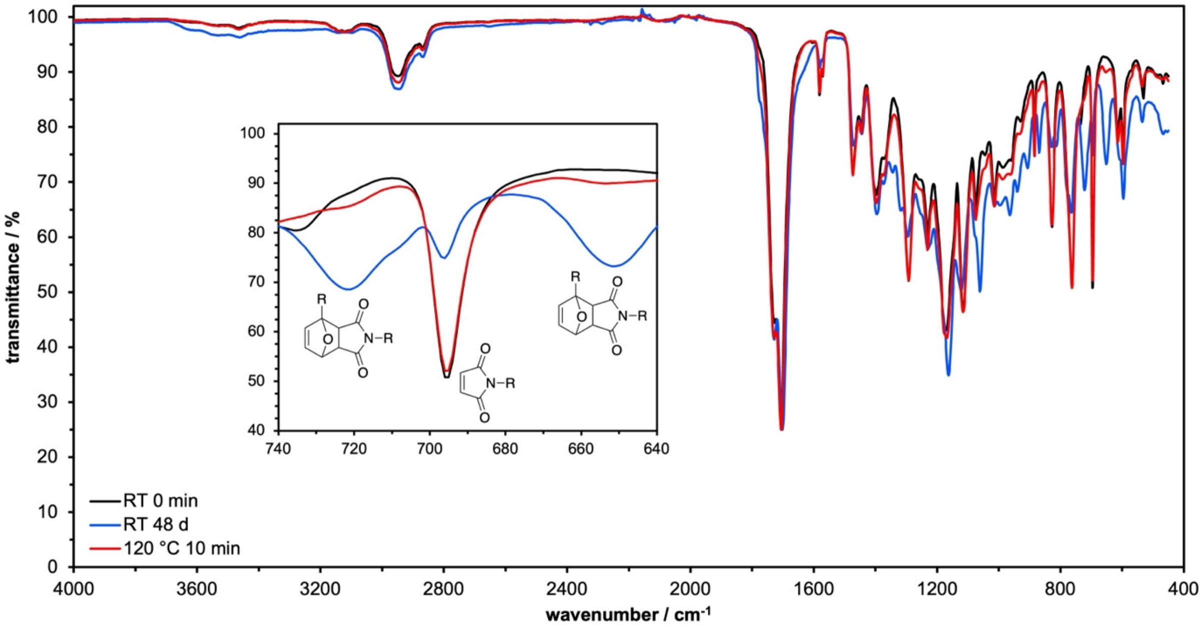

Figure S4. IR-spectra of the furan- and the maleimide-functionalized monomer (1:1). Black: directly after mixing of the monomers. Blue: after crosslinking at for 48 d at RT. Red: after de-crosslinking for 10 min at 120 °C. Maleimide absorption at 696 cm^{-1} is highlighted. Absorption of the cycloadduct occurs at 620 cm^{-1} and 720 cm^{-1}.

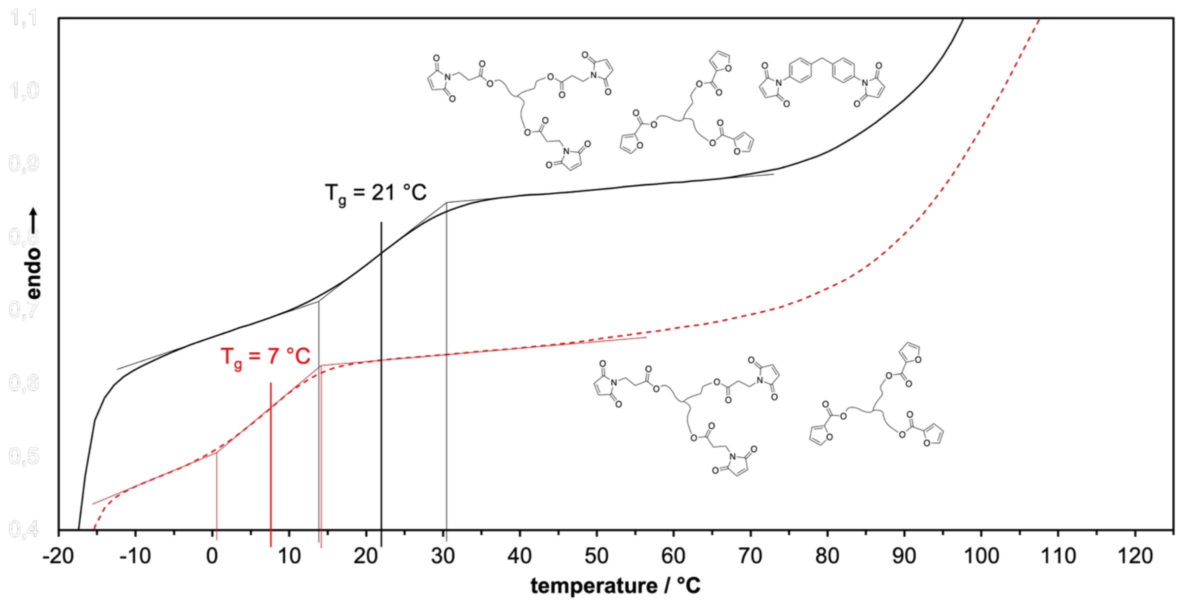

Figure S5. DSC measurement of two cured monomer-mixtures (stoichiometric equivalents in brackets). Dashed red: furan-functionalized monomer / maleimide-functionalized monomer (1:1). Solid black: furan-functionalized monomer / maleimide-functionalized monomer / BMI-S (1:0.9:0.1). Onset and offset are marked, the inflection point was used to determine T_g.

For identification of the crosslinked polymers' T_g, DSC-measurements were performed. **Figure S5** shows two different monomer-mixtures, after curing for 50 days at RT. The first one (dashed red) consisted of the furan- and maleimide-functionalized monomers in a stoichiometric mixture. For the second mixture (solid black), 10 % of the maleimide-functionalities were replaced by the aromatic BMI-S. This yielded an increase of the T_g from 7 °C to 21 °C. At higher temperatures of 80 °C or more, the endothermic signal becomes more distinct, which is attributed to the commencing rDA-reaction.

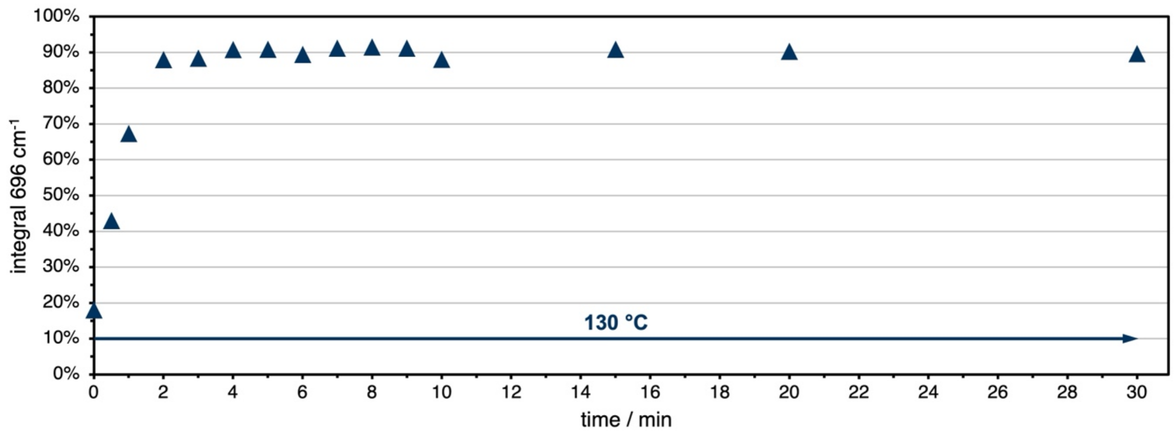

Figure S6. Thermal stability of the mechanically improved mixture of furan- and maleimide-functionalized monomers with BMI-S (1:0.9:0.1) at 130 °C (3rd Cycle, according to Figure 8).
